# Perspectives on use of oral and vaginal antiretrovirals for HIV prevention: the VOICE-C qualitative study in Johannesburg, South Africa

**DOI:** 10.7448/IAS.17.3.19146

**Published:** 2014-09-08

**Authors:** Ariane van der Straten, Jonathan Stadler, Ellen Luecke, Nicole Laborde, Miriam Hartmann, Elizabeth T Montgomery, on behalf of the VOICE-C Study Team

**Affiliations:** 1 Women's Global Health Imperative, RTI International San Francisco, CA, USA; 2 Department of Medicine, Center for AIDS Prevention Studies, University of California San Francisco San Francisco, CA, USA; 3 Wits Reproductive Health and HIV Institute, Faculty of Health Sciences, University of the Witwatersrand, Johannesburg, South Africa

**Keywords:** HIV prevention, antiretroviral, pre-exposure prophylaxis, microbicides, adherence, South Africa, qualitative methods, HIV stigma

## Abstract

**Introduction:**

Antiretroviral (ARV)-based pre-exposure prophylaxis (PrEP) is a promising new HIV prevention strategy. However, variable levels of adherence have yielded mixed results across several PrEP trials and populations. It is not clear how taking ARV – traditionally used for HIV treatment – is perceived and how that perception may affect the use of these products as preventives. We explored the views and experiences of VOICE participants, their male partners and community members regarding the use of ARV as PrEP in the VOICE trial and the implications of these shared meanings for adherence.

**Methods:**

VOICE-C was a qualitative ancillary study conducted at the Johannesburg site of VOICE, a multisite, double-blind, placebo-controlled randomised trial testing tenofovir gel, oral tenofovir and oral Truvada^®^ for HIV PrEP. We interviewed 102 randomly selected female VOICE participants, 22 male partners and 40 community members through in-depth interviews, serial ethnography, or focus group discussions. All interviews were audiotaped, transcribed, translated and coded thematically for analysis.

**Results:**

The concept of ARV for prevention was understood to varying degrees across all study groups. A majority of VOICE participants understood that the products contained ARV, more so for the tablets than for the gel. Although participants knew they were HIV negative, ARV was associated with illness. Male partners and community members echoed these sentiments, highlighting confusion between treatment and prevention. Concerned that they would be mistakenly identified as HIV positive, VOICE participants often concealed use of or hid their study products. This occasionally led to relationship conflicts or early trial termination. HIV stigma and its association with ARV, especially the tablets, was articulated in rumour and gossip in the community, the workplace and the household. Although ARV were recognised as potent and beneficial medications, transforming the AIDS body from sickness to health, they were regarded as potentially harmful for those uninfected.

**Conclusions:**

VOICE participants and others in the trial community struggled to conceptualise the idea of using ARV for prevention. This possibly influenced willingness to adopt ARV-based prevention in the VOICE clinical trial. Greater investments should be made to increase community understanding of ARV for prevention and to mitigate pervasive HIV stigma.

## Introduction

Recently, oral antiretroviral (ARV)-based pre-exposure prophylaxis (PrEP) was established as an effective new HIV prevention strategy. Nevertheless, adequate protection hinges on correct and consistent product use. Adherence challenges have emerged as a key reason for divergent results across PrEP studies, in different locations and with different populations [1–9]. Further, across vaginal and oral routes, daily, intermittent, or coitally related dosing regimens have proved difficult to execute [1, 3, 6, 7, 10–15].

Tenofovir was originally designed for HIV treatment and is part of first-line ARV therapy regimens in Africa. Treatment adherence has been hailed as a success in reducing mortality and morbidity amongst people living with HIV [16–19]. However, public secrecy of HIV status and concealment of AIDS suffering and stigma continue to undermine treatment adherence to ARV; patients may try to avoid revealing their HIV status by concealing their medication, compromising adequate use [20–22].

Similar issues arise with regard to using ARV to prevent HIV acquisition because it may generate social stigma, particularly in contexts where this prevention approach is unfamiliar [23]. In Kenya, stigma was reported to negatively affect participants’ ability to adhere to an oral PrEP regimen [11]. Similarly, Thai participants in another oral PrEP trial feared being mistakenly identified as HIV positive and experienced stigma and relationship stress [14]. Oral PrEP tablets are readily identifiable as ARV and thus attract unwanted public scrutiny [11, 14]. Consequently, participants in prevention studies conceal their tablets because of fears of negative reactions [11, 23, 24], avoid carrying their study products with them [14], or lie about the reason for taking the tablets [11].

The VOICE trial evaluated daily oral and topical tenofovir-based HIV PrEP among women in South Africa, Uganda and Zimbabwe. In September 2011, the oral tenofovir arm (TDF; referred to as “tenofovir” by the study team) was discontinued for futility. In November 2011, a similar determination was made for the tenofovir (TFV) gel arm. The emtricitabine-tenofovir (referred to as “Truvada” by the study team) and oral placebo arms continued until planned exit in August 2012 [6]. All three products tested were found safe; however, none reduced the risk of HIV-1 acquisition, given widespread low product use, based on post-trial pharmacokinetic drug testing in biological specimens [6]. Similar to VOICE, FemPrEP, a phase III trial of oral Truvada^®^ tablets, was unable to demonstrate effectiveness because of low adherence [3].

We recently reported findings from a qualitative study, VOICE-C, which examined the contextual factors influencing daily use of vaginal gel and oral tablets for HIV prevention in the VOICE trial. We found that few participants acknowledged non-use and that social relations within the household and the community shaped women's experiences of trial participation and the trial products [25]. In this paper, we explore further the views and experiences of participants, specifically in relation to the use of ARV as PrEP in the VOICE trial and the implications of these shared meanings for product adherence.

## Methods

VOICE-C was a qualitative exploratory ancillary study implemented between July 2010 and August 2012, concurrent with the VOICE trial. As part of the VOICE trial's procedures (detailed in [6]), when the active products were referred to, staff used a pharmaceutical name (tenofovir or Truvada), or described them as having “medicine.” A number of study materials, as well as education and counselling procedures regularly provided during the trial explicitly stated that the active ingredients in the investigational products contained ARV.

VOICE-C was conducted at one of the 15 VOICE sites, the Wits Reproductive Health Institute (Wits RHI), in Johannesburg, South Africa. The research clinic is located in Hillbrow, a low-income, densely populated, inner-city suburb of Johannesburg. VOICE participants were recruited from Hillbrow, neighbouring suburbs and more distant townships. The VOICE-C study has been previously described [25]. Briefly, VOICE-C enrolled four groups of people: VOICE participants, their male partners, community advisory board (CAB) members and other community stakeholders. The VOICE participants were randomly preselected and randomly assigned to one in-depth interview (IDI; *N*=41) or serial ethnographic interviews (EI; *N*=21) conducted during the course of the VOICE study, or one exit focus group discussion (FGD; *N*=40). Male partners of VOICE participants were recruited to participate in IDI (*N*=14) conducted during the course of the study or an exit FGD (*N*=8) as previously described [25]. Finally, a convenience sample of 40 community members joined FGDs. These included 17 CAB members who participated in up to four serial FGDs and 23 community stakeholders who took part in one of three FGDs, both of which were conducted during VOICE implementation. Each of these latter FGDs comprised individuals who lived or worked in Hillbrow and were homogeneous in terms of their professional affiliation: 1) community-based organisations involved in HIV/AIDS; 2) local media, including community newspapers and radio stations; and 3) a local neighbourhood improvement programme. Out of all participants screened, one female participant, three male partners and one community stakeholder refused participation.

### Procedures

IDIs and FGDs were conducted at the research site. The EIs (a longitudinal series of 2–4 interviews) took place at the interviewees’ home or a private location of her choice. All IDIs, EIs and FGDs were conducted by trained gender-matched research staff in the participants’ languages of choice; they were audio-recorded, transcribed and translated into English (when conducted in another language). The interviews included a short demographic questionnaire. Selected survey data were collected during participants’ VOICE clinic visits.

**Figure 1:**
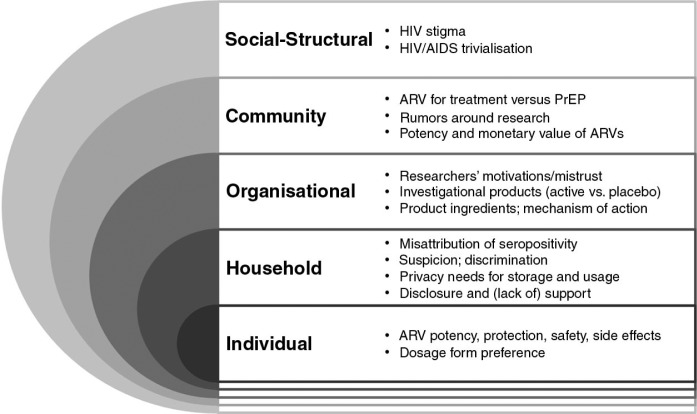
Socio-ecological framework of factors affecting perceptions about ARV for pre-exposure prophylaxis (PrEP), by levels of influence, among VOICE-C participants. Note: Some of the factors may operate at multiple levels of influence, but are only presented at the highest level at which they operate and are not repeated in lower levels. The organisational level of influence in this framework is focused on the clinical trial setting.

All English language transcripts were coded in Nvivo (version 9.0, Burlington, MA) by the analysis team, which included members of the data coordinating centre and site staff, using a codebook that followed a socio-ecological framework (SEF) (Figure 1) [25]. Coded data were concatenated into coding reports by thematic area (e.g., ARV, PROTECT, SIDE EFFECT, PREFERENCE) and by SEF level codes (e.g. HOUSEHOLD, CLINIC, COMMUNITY) and then summarised into memos. Memos from each area were further analysed to reveal patterns or themes related to the use of ARV in the trial. The study team was blinded to study arm assignments of VOICE participants until the last analytical step, when participants’ perceived product assignment was assessed.

All participants provided written informed consent prior to participation. The study protocol was approved by the Institutional Review Boards at RTI International and the University of the Witwatersrand, and overseen by the regulatory infrastructure of the U.S. National Institutes of Health and the Microbicides Trial Network.

## Results

Of the 102 ethnically diverse VOICE female participants who joined VOICE-C, the mean age was 27 years, 68% had completed secondary school and 96% had a main male partner although only 22% were married. Women completed a structured survey at VOICE trial exit asking about their perceived product assignment. Although 51% were actually assigned to an active treatment arm, 62% perceived having received an active product (Table 1). The characteristics of the other groups in VOICE-C, the male partners (*N*=22) and the community members (*N*=40), are also presented in Table 1.

**Table 1:** Demographic characteristics of participants by VOICE-C study groups

	VOICE participants	Male partners	Community members
At time of VOICE-C (first) interview	*N*=102 (%)	*N*=22 (%)	*N*=40 (%)
Median age (mean, range)	26.8 (19–40)	31.4 (22–45)	38.5 (20–60)
Currently married	22 (22%)	9 (41%)	17 (43%)
Has current primary sex partner	98 (96%)	22 (100%)	
Length of relationship in years (mean, range)	5.5 (0.1–25)	5.9 (1–10)	
Currently living with primary sex partner	44 (43%)	16 (72%)	
Completed secondary school or more	69 (68%)	14 (64%)	31 (78%)
Does not earn an income	44 (43%)	4 (18%)	13 (33%)
Ethnic group			
Zulu	27 (26%)	4 (18%)	5 (13%)
Xhosa	13 (13%)	3 (14%)	5 (13%)
Sotho	19 (19%)	4 (18%)	10 (25%)
Ndebele	26 (25%)	5 (23%)	1 (3%)
Other^a^	17 (17%)	6 (27%)	19 (48%)
Religion			
Christian	94 (92%)	17 (77%)	36 (90%)
Muslim	0 (0%)	2 (9%)	0 (0%)
Other/none	8 (8%)	3 (14%)	4 (10%)
History of involvement with HIV research/work	43 (42%)	5 (23%)	31 (78%)
Type of interviews received^b^			
In-depth interview	41 (40%)	14 (64%)	
Ethnographic interview	21 (21%)		
Focus group discussion	40 (39%)	8 (36%)	40 (100%)
(Initial) interview conducted prior to first DSMB	44 (43%)	9 (41%)	28 (70%)
Treatment arm assignment			
Truvada^®^	22 (22%)		
Viread^®^	18 (18%)		
Oral placebo	22 (22%)		
Tenofovir (TFV) gel	21 (21%)		
Placebo gel	19 (19%)		
Perceived assignment to active product (*N*=83)^c^	53 (64%)		

a Other ethnic groups: KALANGA 1, KHALANGA 1, NYANJA 1, SHONA 2, SWATI 1, SWAZI 1, TSONGA 3, TSWANA 4, VENDA 3

b the procedural changes because of VOICE Data Safety and Monitoring Board (DSMB) futility recommendations contributed to several women randomly preselected for VOICE-C participation receiving an earlier interview than anticipated, being reallocated to a different interview modality, or to stopping their serial ethnographic interviews early

c perceived product assignment was assessed at the VOICE clinic exit visit, TDF participants (*N*=18) were not asked this question, because of early stopping of this arm following the first DSMB futility recommendation [26]. IQR=inter quartile range.

### ARV for treatment versus prevention

VOICE-C participants had varying levels of awareness that active trial products contained ARV, although there was greater cognizance that the tablets contained ARV than the vaginal gel. Among gel participants, about half were unaware that the active gel contained ARV. Participants were initially surprised when they learnt that the trial was testing ARV as a means of preventing HIV. Lily (all names are pseudonyms) recounts her feelings upon such learning:

I didn't have a problem with the tablets but I only got scared when I heard that these tablets are ARV, I got scared to think that we are now going to take tablets used by people who are sick when we weren't sick at all. (Lily, age 40, Tablet FGD)

In some cases, women speculated that the products only contained “some of the same components as ARV for treatment.” Others such as Limpho initially misunderstood the information provided by staff at the trial clinic.

(…) According to me, they said they are doing a research to find out if they [ARV] work, you see, but then in my mind I thought, okay, maybe it will help protect people that have AIDS. I had not understood correctly. (Limpho, age 21, Tablet EI)

Otillia used the acronym ART (antiretroviral therapy) to differentiate the study drugs from ARV, in a conversation with her friend.

I showed her and she said “Yoh! They look like ARV,” and I said “Yes they look like this but at the clinic they said these study tablets are ART they are not the same as ARV,” you see? (Otillia, age 26, Tablet EI)

When the interviewer prompted Otillia about the difference between ARV and ART, she first said they are different “because of what they do in the body,” but ended up confused with her own explanations.

However, several participants reacted negatively. Thoko (Gel, age 25, EI) stated that she would not use the gel if it contained ARV and would drop out of the study. According to her, “ARV are for sick people” and not for prevention. She did not understand the reasons to give ARV to healthy women.

The uncertainty and confusion expressed by many participants like Thoko stems partly from difficulties in distinguishing between treatment and prevention, particularly relating to using ARV. IsiZulu and SeSotho speakers used the words *ukuvikela* or *sirelela*, respectively, for prevention or treatment, while others used the English words *treat* and *prevent* interchangeably. With respect to ARV taken for treatment, participants also used the English word *cure*. The conflation of treatment and prevention potentially creates difficulties in conceptualising the idea of PrEP, particularly when the prevention technologies use the same ARV drugs, usually taken for treatment. Indeed, in some cases, HIV-positive family members stole participants’ tablets, presumably to treat their illness. Palesa's (Tablet, age 21, FGD) mother acquired a sexually transmitted disease and, worried that she may also be infected with HIV, started using her daughter's trial tablets. Tshepiso's (Tablet, age 27, FGD) aunt was HIV positive and stole her tablets when her own ARV ran out.

Male partners were not enrolled in the VOICE trial and unsurprisingly did not have a clear understanding of the trial in general or of using ARV for prevention in particular. Some questioned the legitimacy of the trial when they learnt that ARV were tested: for example, they asked why HIV-negative women received ARV and suspected that their female partners may be HIV positive because they used ARV.

Other male partners were more concerned about the health risks associated with ARV [27]. One man said his partner may acquire HIV from taking ARV and that this would affect their future progeny. Although he insisted that his partner cease taking the trial tablets and end trial participation, she remained. Others were more sympathetic, although they too were poorly informed. For example one man thought the study was meant to detect ARV in different biological specimens and trusted that medical researchers would not give his partner unsafe drugs. Despite the negative associations of the tablets, male partners appeared to prefer these to the gel, because of the unwanted vaginal wetness that could occur after inserting gels.

The CAB and other stakeholders articulated their difficulties in communicating the concept and providing the correct information about ARV for prevention in the community. They also expressed concerns with the side effects of PrEP, and that women in VOICE would be identified as HIV positive. Nevertheless, they said that PrEP would be acceptable if it was found to be efficacious in preventing HIV acquisition.

### PrEP and HIV stigma

All categories of participant (VOICE participants, male partners and community members) recounted overarching apprehensions with HIV stigma in their community. VOICE participants sometimes referred to HIV/AIDS as “that disease” and “the flu” and one remarked that “being HIV positive is seen as being in style” (Mandisa, age 25, Gel IDI). This appears to downplay or trivialise the seriousness of the disease, particularly in the era of effective treatment. As one male partner stated:

People they still die before knowing about AIDS and people are still dying, they are still going to die, so what's so special about this AIDS, you understand? That's what they think. If I get HIV/AIDS I will just go and get treatment and live longer than those people who are not even infected, so why worry. (Antonio, male partner FGD, Gel)

Yet, it also signifies a widespread view of the ubiquity of the HIV/AIDS epidemic and the futility or hopelessness in trying to prevent its spread.

Local constructions of HIV such as these had implications for women who used ARV to prevent HIV and feared being mistakenly identified as HIV positive and suffering discrimination and social isolation. Trial participants recognised this and went to great lengths to disguise their involvement in VOICE. They were secretive and hid their products from household members, co-workers and friends to avoid accidental disclosure, although this made taking the products more difficult. Keneoe (Gel, age 21, EI) hid her gels in the ceiling to avoid detection and Tale applied her gel in the toilet, the only private place in her home.

At times there will be a lot of people in the house and you know how it is like in South Africa. The bedroom serves a multi-purpose task of being a sitting room at the same time. Hence the bathroom is always the safer option where I would be able to do my business [insert gel] without disturbance from anyone. (Tale, age 24, Gel IDI)

For trial participants, explaining the reasons for taking the tablets was often too burdensome because it was easier to be secretive. “I hated that thing of having someone ask me because I would have to explain everything,” said Nomsa (Tablet, age 26, FGD).

Conscious of the possibility of unkind gossip and rumour, and of discriminatory action being taken against her, Naledi remarked:

The area that I live in has a bad influence because they like to gossip about people. If they could see these tablets, obviously they are going to gossip (…) if they could see me taking the tablets they will say that I have AIDS. They would not listen to my explanation about these tablets. (Naledi, age 23, Tablet IDI)

For some, the risk of being labelled HIV positive outweighed the benefits of participating in the trial. Gladys experienced discrimination first hand when her flatmates suspected she was HIV positive:

There is that stigma, discrimination and stigmatisation of those people who have got AIDS. So, they [flatmates] started to change the way in which they were living, you know. When we have drunk with this cup, they will just not touch it – they will start doing those things. (Gladys, age 33, Tablet EI)

This had implications for adherence and participation in the trial. Nogoli (Gel, age 31, FGD) recounted a study participant who withdrew from the trial because her mother-in-law pressured her to stop taking ARV by saying “you cannot take ARV when you are not sick. They [researchers] only want to make you sick.”

However, there are also several examples of women who managed these outsiders’ perceptions by referring to the VOICE trial fact sheets, undergoing HIV testing at a local clinic with suspecting relatives, or simply discounting the disparaging remarks made of them as a result of ignorance; such is the case for Thuli's cousin:

She says I will catch the flu [HIV]. I tried to give her the papers [fact sheets] to read and see what this thing [VOICE] is all about is. She is ignorant and she pretends that she knows everything. […] She said that if I continue testing I will be infected. (Thuli, age 24, Gel FGD)

Overall, VOICE participants assigned to gel preferred it to the tablet, while tablet users were divided about their preference for tablets or gel. The association of study products with ARV was seldom specifically mentioned when stating product preference. However, six women did invoke this as a reason to prefer gel over tablets: gel did not carry the HIV stigma associated with the tablets, and is less recognisable, as people don't know what the gel is for.

### ARV potency and safety

Participants’ ambivalence towards ARV was expressed several ways: on the one hand they recognised that ARV are extremely potent, transforming the AIDS body from sickness to health, and were therefore highly beneficial.

ARV are taken by positive people, they get fat isn't it? That thing encourages me because I live with positive people in my community. I see how the ARVs are working on them. So that thing encourages me to want to take them. (Javas, age 30, Tablet FGD)

This awareness of the power of ARV influenced participants’ accounts of the side effects. For example, akin to the dramatic physical transformations of HIV-infected people on treatment, many tablet participants reported weight gain, which they attributed to taking the tablets (although there was no evidence of this in the clinical data). Others regarded the side effects as confirmation that they had been randomised to the active arms of the study products, whereas those who did not experience any side effects tended to think they had been randomised to placebo. Yet, the potency of ARV was also regarded as a source of potential harm for those uninfected.

I did have my concerns with these things since well it is part of the ARVs and you know what the ARVs does in your body[…] Even today I know that ARVs can react badly in your body so this [gel] in the long run, will it not affect me? (Abri, age 31, Gel IDI)

There also was some speculation amongst community members and male partners that taking ARV could cause one to seroconvert. Stories that circulated in the media and amongst trial participants about ARV sold and stolen to mix in with a heroin or crystal methamphetamine-based drug called *Nyaope* (also known as *Whoonga* or *Wunga*) added to the popular conceptualisation of the dangerous potency of ARV [28].

I do not feel safe since there are people in the community who smoke ARVs to get high. Thugs mix ARVs with some other things […] I heard that the [thugs] sell ARVs and they get good profit. (Jewel, age 24, Tablet IDI)

These rumours added another dimension to the stories of ARV potency and danger – that of physical risk – associated with carrying ARV in public places.

## Discussion

This qualitative study conducted in Johannesburg, South Africa, explored local perspectives on using ARV as PrEP to prevent HIV in the VOICE trial, which was unable to demonstrate effectiveness because of low product adherence. Focusing our analysis on the social meanings of ARV, we found a variety of perspectives amongst trial participants, their male partners and community members. Recognition that the study products contained ARV was not unanimous and women's understandings of PrEP were mediated through confusion surrounding the use of ARV as a prevention method, versus treatment. Understandings of health that were articulated here were often incommensurate with biomedical models, revealing a tendency to conflate prevention and treatment. Yet, the very fact that many participants knew ARV are used for treatment, inferred a known distinction between prevention and treatment. This apparent contradiction may stem from the fact that ARV for prevention was difficult to grasp and reconcile with the more familiar treatment model. In other words participants had trouble distinguishing ARV for prevention from treatment and yet were aware that it wasn't the same thing.

Given high HIV prevalence and stigmatisation of the disease, taking ARV for prevention became a marker of HIV infection. Despite a few participants suggesting that HIV seropositivity may be normalized or even trendy, the strong association of ARV with a positive status threatened to engender HIV stigma and secrecy around the use of the trial products and potentially challenged product adherence and trial participation. Although trial participants had worries about side effects, concerns about the social impact of being identified as HIV positive appeared to be greater. The stigma of HIV is based not only on sexual morality, but also on the perception of HIV as an incurable illness [29] and has contributed towards the secrecy and concealment of serostatus [30]. The threat of a “spoilt identity” [31] from being mistakenly identified as HIV positive influenced participants’ willingness to use ARV for prevention, especially in the context of a placebo-controlled trial where the actual benefit of using these products was uncertain.

The novelty of PrEP for HIV prevention combined with pervasive HIV stigma [32, 33] led to anxieties about being identified as HIV positive, more so among tablet than gel participants. Although ARV were recognised as having a positive transformative effect on AIDS sufferers [34], their potency was also regarded as dangerous, particularly when used by individuals who were not HIV positive.

As growing numbers of clinical trials are conducted in settings where there is limited experience of biomedical research practices, the need for understanding local constructs of disease and responses to novel medical technologies increases. How are these new applications of drugs such as ARV understood and negotiated in contexts that are characterised by stigma and blame, and what are the implications for wider access to PrEP in mainstream public health care? In this study we found that ARV for prevention was interpreted with ambivalence. Although regarded as life-saving medications, ARV were also seen as potentially harmful; hence the belief, albeit not widely shared, that ARV taken by uninfected individuals could lead to acquiring HIV, or the association of ARV with illegal narcotics and criminality. The result of this ambivalence created uncertainty and doubt, potentially undermining women's motivation to adhere to the trial products.

Other studies of HIV prevention interventions have reported challenges with local understandings of biomedical models [35]. A potentially fruitful area of social research may be how biomedical researchers can engage with local understandings of prevention, treatment and cure to improve correct usage and sustained adherence to ARV for PrEP in southern Africa and elsewhere.

Two seemingly opposite undercurrents of opinions emerged here: one linked to HIV stigma and the other to ubiquity and trivialisation of HIV/AIDS. Although South Africa is a country most heavily burdened by the AIDS pandemic, the relatively recent universal access to ART has changed the landscape, turning a terminal disease into a chronic, manageable illness. Simultaneously, stigma and discrimination against those infected persist and prevent HIV-positive individuals from accessing the treatment and care they need [36]. Although these perceptions appear to go in different directions they may both work against acceptance of ARV for PrEP: one because prevention is no longer seen as important and the other because misattribution of seropositivity may discourage those at risk to access or use ARV for PrEP.

There are several limitations to this study: VOICE was implemented in three countries and across 15 sites, VOICE C was conducted at only one site, in Johannesburg, thus our findings may not reflect the perspectives of participants at other sites. Further, in South Africa, ARVs have been the centre of controversy, particularly under the leadership of former President Thabo Mbeki and his Health Minister, Manto Tshabalala-Msimang, who publicized the alleged toxic effects of ARV, and questioned the orthodox view of HIV causing AIDS [37]. This may have uniquely shaped VOICE-C participants’ experiences regarding both mistrust of research and ARV for prevention. Given South Africa's central place in the global AIDS pandemic, this warrants further research. Notably, studies in South Africa and other countries corroborate our findings that taking “AIDS pills” or ARVs for prevention generates concerns and may be associated with product non-use [11, 23, 24, 38].

In summary, socio-contextual factors influenced willingness to adopt ARV-based prevention, in the context of a large clinical trial. Greater investments should be made to increase community-wide understanding of ARV for prevention and to mitigate pervasive HIV stigma.
